# Application and design considerations of ROS-based nanomaterials in diabetic kidney disease

**DOI:** 10.3389/fendo.2024.1351497

**Published:** 2024-04-29

**Authors:** Qing Huang, Jiahao Tang, Yunchuan Ding, Fangping Li

**Affiliations:** Department of Endocrinology, The Seventh Affiliated Hospital, Sun Yat-sen University, Shenzhen, China

**Keywords:** reactive oxygen species (ROS), nanomaterials, diabetic kidney disease, treatment, design considerations

## Abstract

Diabetic nephropathy (DKD) is a common chronic complication of diabetes mellitus and an important cause of cardiovascular-related death. Oxidative stress is a key mechanism leading to diabetic nephropathy. However, the current main therapeutic approach remains combination therapy and lacks specific therapies targeting oxidative stress. With the development of nanotechnology targeting ROS, therapeutic fluids regarding their treatment of diabetic nephropathy have attracted attention. In this review, we provide a brief overview of various ROS-based nanomaterials for DKD, including ROS-scavenging nanomaterials, ROS-associated nanodelivery materials, and ROS-responsive nanomaterials. In addition, we summarize and discuss key factors that should be considered when designing ROS-based nanomaterials, such as biosafety, efficacy, targeting, and detection and monitoring of ROS.

## 1 Introduction

Diabetic kidney disease (DKD) is the most severe microvascular complication of diabetes, constituting a predominant cause of end-stage renal disease (ESRD) in many developed countries (1). However, the current prevention and treatment strategy of DKD mainly aims to control blood glucose and blood pressure comprehensively. While employing renin–angiotensin–aldosterone system antagonists and glucose cotransporter-2 inhibitors has demonstrated efficacy in retarding the initiation of DKD, a significant subset of patients still exhibits a continuous trajectory of renal deterioration (2, 3). Moreover, many small molecule drugs for DKD usually require high concentrations, which may lead to non-specific uptake and adverse side effects. Therefore, specific treatments for DKD should attract our attention (4).

Many factors are believed to contribute to the development of DKD, with oxidative stress being an essential pathological mechanism. Moreover, hyperglycemia is a primary reason for promoting excessive reactive oxygen species (ROS) production in oxidative stress (5). Not only do ROS orchestrate cellular equilibrium, but they also serve as primary instigators of cellular aberrations, thus accentuating disease manifestations (6). For individuals diagnosed with DKD, ROS production surges, overpowering the neutralizing capabilities of antioxidants. This ensues a precarious imbalance between pro-oxidative and antioxidative mechanisms, culminating in oxidative stress, amplified apoptosis, and consequential renal fibrosis (7). Hence, strategies focusing on mitigating oxidative stress and neutralizing excessive ROS present promising avenues for DKD management. Given the nanotechnological advancements, many nanomaterials endowed with unique ROS modulatory properties have been synthesized (8). These ROS-centric nanomaterials can either stimulate or mitigate ROS production, aiming for therapeutic modulation.

In recent years, nanotechnology has experienced significant advancements in the field of targeted tumor therapy. Nanoparticles (NPs) have emerged as versatile tools that can effectively modulate the levels of reactive oxygen species (ROS) and encapsulate a diverse array of therapeutic agents, including small molecule drugs, peptides, and nucleic acids. These nanoformulations enable the sustained and controlled release of therapeutic payloads *in vivo*, offering enhanced precision and efficacy in cancer treatment (9). NPs as drug carriers have many advantages, such as high stability (i.e., long shelf life), high carrier capacity (many drug molecules can be added to the particle–matrix), and capability to deliver both hydrophilic and hydrophobic substances through different routes such as oral administration, inhalation, and so on. These carriers can also be designed to enable controlled (sustained) release of drugs from the matrix (10). Furthermore, tailoring parameters like size, morphology, charge, and surface properties of these nanoscale delivery systems, keeping in view the physiological or pathological nuances of the kidney, can enhance kidney-centric therapeutic interventions (11).

In this review, we discussed and summarized the applications of different types of ROS-based nanomaterials in DKD, including ROS-scavenging nanomaterials, ROS-related nanodrug delivery materials, and ROS-responsive nanomaterials. Common applications in current research are highlighted. In addition, to give full play to the role of ROS nanomaterials, we also summarized the issues that need to be considered in the preparation of ROS nanomaterials. We hope this short review can provide some valuable hints and references for the development of more effective and safer ROS-based nanomaterials in the future.

We searched PubMed using the keywords “diabetic kidney disease,” “ROS,” “kidney disease,” and “nanomaterials” to obtain current relevant literature. Literature related to nanomaterials targeting ROS-related mechanisms for the treatment of diabetic kidney disease and other kidney diseases is discussed, while nanomaterials targeting other pathogenic mechanisms are not discussed. In addition, since there is a large body of literature on the use of ROS for the treatment of other renal diseases, which is not the endpoint of this review, only a few references are cited to elaborate on them.

## 2 Different types of ROS-based nanomaterials for treating DKD

### 2.1 ROS-scavenging nanomaterials

#### 2.1.1 Characteristics of ROS-scavenging nanomaterials

**Figure 1:**
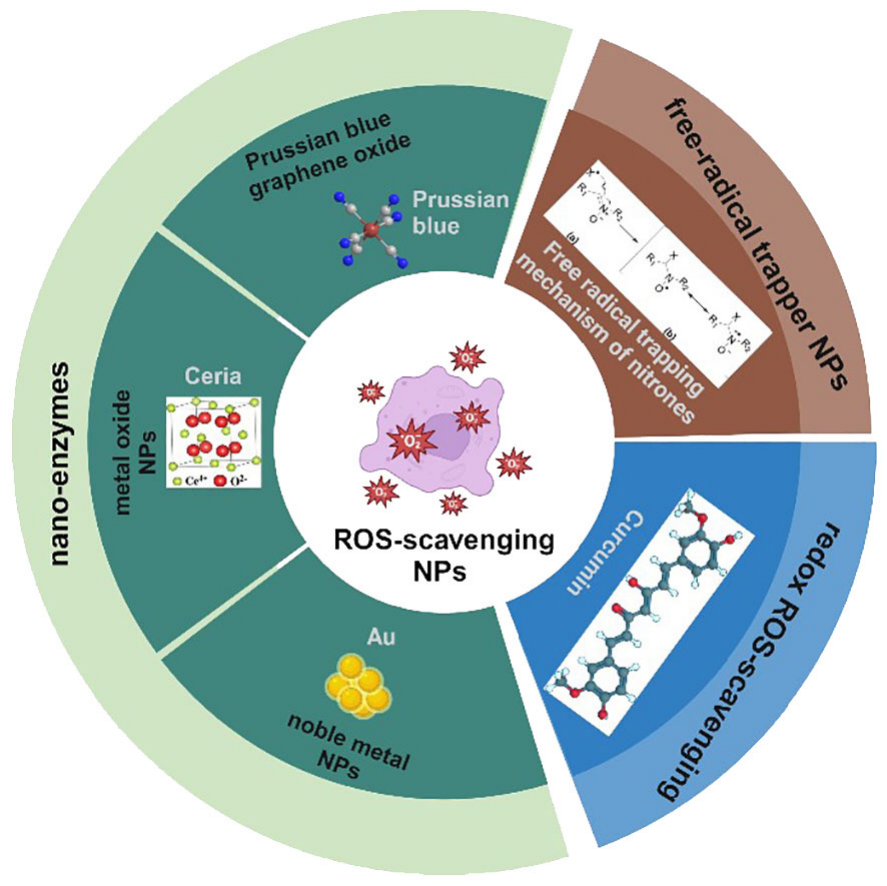
Classification of ROS-scavenging NPs.

ROS-scavenging nanoparticles can be broadly categorized based on their operative mechanisms into three distinct classes (12): enzyme-mimicking nanoparticles (nano-enzymes), free radical trapper nanoparticles, and redox ROS-scavenging nanoparticles. Common enzyme-like NPs include noble metal nanoparticles [Au, Ag, Cu (13–15), etc.], metal oxide nanoparticles [CeO_2_, Fe_3_O_4_, MnO_2_ (16–18), etc.], Prussian blue graphene oxide (19), etc. These nanoparticles typically exhibit multi-enzyme activities akin to peroxidase (POD), catalase (CAT), and superoxide dismutase (SOD), facilitating ROS neutralization. While SOD typically catalyzes the decomposition of O_2_^−^ to H_2_O_2_, POD and CAT catalyze the decomposition of H_2_O_2_ into water. Free radical trapper NPs capture ROS via the single electron on nitroxide or conjugated double bonds (20, 21). The primary mechanism of redox ROS-scavenging NPs is related to the stimulation of redox activity by targeting various substances, thus achieving the purpose of scavenging ROS (22, 23) (**Figure 1**).

In designing the removal of ROS nanomaterials, we need to consider preventing leakage of the remover. If antioxidants are prematurely released from nanocarriers before arriving at the intended site, they may culminate in diminished therapeutic efficacy or even engender adverse reactions (24). Some studies have shown that the drug delivery platform based on nanomaterials can be stabilized by enhancing the molecular interactions, such as hydrophobic interaction, electrostatic interaction, van der Waals force, hydrogen bond, covalent bond, and the encapsulation between drugs and nanomaterials (25, 26). Moreover, enhancing the active-targeting proficiency of nanomaterials is imperative. Active targeting based on ligand–receptor recognition may show better efficacy than passive targeting in human cancer therapy, and several active-targeting nanomedicines have progressed into clinical trials (27). Active targeting is based on the recognition and binding of ligands to cell surface receptors, and they can accomplish site-specific delivery by modifying their surface ligands, which can recognize overexpressed receptors in pathological tissues, thereby promoting the selective uptake of nanomedicines (28). Introducing specific stimuli within the antioxidant delivery system can curtail the premature release of encapsulated antioxidants. Notably, the prime merit of stimulus-responsive release systems lies in their spatial–temporal control of localized drug release, offering a promising mechanism for drug release activation.

#### 2.1.2 Application of ROS-scavenging nanomaterials

Several ROS-scavenging nanomaterials have been investigated for their potential therapeutic effects on DKD. Alomari and his team (29) explored the therapeutic efficacy of 50-nm gold nanoparticles (AuNPs) in treating DKD. Diabetic rats were treated intraperitoneally with 50 nm of AuNPs (2.5 mg/kg) daily for 7 weeks. Their findings substantiated the robust therapeutic potential of AuNPs in DKD management. The AuNPs exhibited noteworthy preventive capabilities against hyperglycemia-induced detrimental effects in renal tissue, mainly by mitigating the accumulation of extracellular matrix proteins and ameliorating podocyte injuries. This was achieved by suppressing growth factors, inflammation, and angiogenesis markers. Remarkably, AuNP treatments led to a significant reduction in renal oxidative stress, as evidenced by enhanced activities of renal SOD and CAT and a decreased malondialdehyde (MDA) tissue level. These outcomes align with preceding *in-vivo* investigations that attributed antioxidative and antihyperglycemic properties to AuNPs in diabetic animal models.

In another study, Tong and associates (30) formulated a hollow mesoporous silica nanocomposite (HMSN) particle infused with trace cerium oxide by coprecipitation. This nanocomposite demonstrated capabilities in averting ROS-induced DKD pathology and showcased an impressive drug-loading capacity. The nanoparticles were infused with metformin (MET), culminating in the creation of multifunctional nanoparticles (MET-HMSN-CeO_2_). DKD rats were treated with intraperitoneal administration of MET-HMSN-CeO_2_ daily for 8 weeks. The cyclical transition between Ce^3+^ and Ce^4+^ within this system’s mixed-valence ceria augments its antioxidative potential by extending ROS-scavenging activity. The therapeutic potential of these nanoparticles was validated on a streptozotocin-induced renal injury rat model and a high-glucose-induced NRK-52E cell line. The findings underscored their potential to alleviate DKD symptoms through oxidative stress reduction, inhibition of cellular apoptosis, and renal injury protection in both *in-vitro* and *in-vivo* settings. Building on this line of inquiry, Iftekhar Hassan et al. (31) delved into the therapeutic merits of selenium nanoparticles (Se-NPs) with inherent antioxidant properties in addressing nephropathy in the progeny of diabetic rats. The study found that Se-NPs could modulate oxidative stress and inflammation-triggered diabetic conditions in maternal rats, shielding their offspring from diabetes and its associated nephropathy. These research findings underscore the immense potential of targeted removal of excessive ROS in the treatment of DKD.

### 2.2 ROS-related nanodrug delivery materials

Apart from nanomaterials exhibiting ROS-scavenging capacities, there are drugs endowed with antioxidant potential that, when combined with nanoparticles, can amplify both the drug’s target specificity and its stability. Manna and coworkers (32) pioneered the synthesis of AuNPs using pomegranate peel ellagitannins (PPE-AuNPs). The pomegranate peel extract (PPE) is rich in polyphenolic compounds like flavonoids and condensed and hydrolyzable tannin variants, exhibiting pronounced antioxidant activity through their free radical-scavenging mechanisms. Yet, these bioactive molecules inherently face stability issues and are susceptible to spontaneous enzymatic hydrolysis. By constructing PPE-AuNPs, these bioactive entities are rendered more stable. Diabetic mice were treated with different concentrations of PPE-AuNPs via intraperitoneal injection. Studies revealed that PPE-AuNP could holistically overturn the oxidative milieu, curbing protein glycation, scavenging ROS, and deactivating the MAPK/NF-κB/STAT3-induced inflammatory stress. Importantly, PPE-AuNPs were observed to invoke an endogenous antioxidant response, stabilizing renal homeostasis in DKD. Moreover, Tong et al. (33) designed a nanoparticle complex by crafting the poly(ethylene glycol)-block-(poly(ethylenediamine 1-glutamate)-graft-poly(ϵ-benzyloxycarbonyl-1-lysine)) (PEG-b-(PELG-g-PZLL)) carrier loaded with quercetin, abbreviated as QUE/PEG-b-(PELG-g-PZLL) or QUE/P by a previous method (34). Quercetin, a plant flavonoid, is recognized for its formidable *in-vitro* antioxidant characteristics. Emerging research posits quercetin as a potent countermeasure against DKD. Nonetheless, its hydrophobicity, suboptimal bioavailability, and transient half-life impede its *in-vivo* application. The constructed PEG-b-(PELG-g-PZLL) showed potential in elevating the serum quercetin concentration in Sprague–Dawley rats, extending its circulatory lifespan. SD rats were administrated a dose of 10 mg/kg of QUE/P via abdominal subcutaneous injection. In the DKD rat model, both QUE and QUE/P demonstrated remedial effects, evident from renal recovery, metabolic modulation, and curtailed oxidative stress, denoted by reduced MDA levels and augmented SOD activity. Lastly, Akram Ahangarpour and associates (35) inspected the remedial efficacy of myricitrin and its solid lipid nanoparticles (SLNs) vis-a-vis DKD instigated by streptozotocin-nicotinamide (STZ-NA) in mice. Myricitrin, a pivotal flavonol glycoside, showcases potent counteractions against elevated glucose, oxidants, inflammation, and apoptosis. Yet, its oral uptake efficacy remains subpar. In this context, SLNs emerge as an avant-garde nanodelivery mechanism, bolstering the oral bioavailability of flavonoids. In tandem with its SLN version, indications suggest that myricitrin offers tangible improvements in DKD by mitigating oxidative strain and augmenting antioxidant enzyme levels in diabetic mice treated with SLN. These nanomaterials enhance drug stability and bioavailability by loading drugs with antioxidant effects and treat DKD by exerting their antioxidant activity.

#### 2.3 Others

Beyond their capacity to combat oxidative stress, ROS-responsive nanomaterials also present promising therapeutic prospects for treating DKD. These nanomaterials are primed to react to elevated ROS concentrations at the site of the lesion, initiating targeted drug release (36). Such materials have found substantial utility in oncology treatments. Contemporary nanomedicine has embraced ROS-responsive functional groups like sulfur ether, peroxalate ester, and thioketal groups, all of which can undergo cleavage at high ROS levels (37). Yet, as an emergent therapeutic strategy targeting the kidneys, these materials’ *in-vivo* degradability and potential nephrotoxicity need to be more adequately explored. There needs to be more research on applying these nanomaterials specifically to DKD, underlining the need for more comprehensive studies in this area.

## 3 ROS-based nanomaterials for treating other kidney diseases

In addition, we explored other kidney-targeted nanoparticle systems that have been applied to non-DKD. Based on the pathogenesis of chronic kidney disease (CKD), mitigating excessive ROS and restoring the redox equilibrium emerge as pivotal therapeutic strategies. Adhikari and coworkers (38) delved into the therapeutic potential of spinel-structured, citrate-functionalized Mn_3_O_4_ nanoparticles (C-Mn_3_O_4_ NPs) as a redox remedy against CKD. There is existing evidence suggesting that C-Mn_3_O_4_ efficiently catalyzes free radical reactions *in vitro*, particularly those involving hydrogen peroxide. Furthermore, within the hausmannite structure of C-Mn_3_O_4_ NPs, the dynamic interplay among Mn^3+^, Mn^4+^, and Mn^2+^ is crucial for sustaining a redox balance. This research underscored the salience of a nanoparticle’s redox regulatory activity in CKD treatment both at the cellular and organismal scales. Their findings showed that C-Mn_3_O_4_ NPs can redress the redox imbalance in HEK293 cells under H_2_O_2_-evoked oxidative stress, bolster the cellular antioxidant defense arsenal, and mitigate damage to the glomeruli and tubulointerstitial structures in CKD-afflicted mice. The ability of C-Mn_3_O_4_ NPs to balance the redox state has certain potential applications in the treatment of DKD.

Ni and coworkers (39) introduced molybdenum-based polyoxometalate (POM) nanoclusters, innovative nano-antioxidants geared toward kidney protection. Notably, POM tends to accumulate in the kidney over extended durations (40). Under specific redox conditions, the Mo ions in POM fluctuate between Mo^5+^ and Mo^6+^ valences, positioning them as effective ROS scavengers (41). Furthermore, in certain reducing and oxidizing conditions, Mo ions in POM exhibit variable valence between Mo^5+^ and Mo^6+^ and can scavenge harmful ROS (42). This study further demonstrated the efficient ability of POM nanoclusters to scavenge ROS *in vitro*. In other acute kidney injury (AKI) mouse models, renal function in AKI mice was improved when treated with POM nanoclusters, and the dynamic curves of ^68^Ga-EDTA in the blood pool, kidneys, and bladder were almost identical to that of normal mice, indicating that POM nanoclusters can scavenge ROS and produce therapeutic effects. The protective effect of POM nanoclusters against AKI in animal models suggests their therapeutic potential in treating AKI and other ROS-related diseases. Furthermore, Nagasaki and his colleagues designed pH-responsive NPs containing nitrogen oxide radicals to act as ROS scavengers, effectively alleviating AKI (43). This shows that direct removal of excess ROS is one of the effective treatments for AKI.

These nanomaterials have shown remarkable effects in dealing with ROS production and clearance, and although current research models do not focus on DKD, their ability to target kidneys and balance REDOX and antioxidant stress gives them some potential in treating DKD. In future studies, we believe that more in-depth research and systematic comparative analysis are needed to select the most suitable nanoparticle system for DKD therapy. Developments in this area will provide strong support for more effective and safer treatments for DKD.

## 4 Design considerations of ROS-based nanomaterials

The serendipitous discovery of nanomaterials’ renal targeting and selective accumulation has opened new therapeutic avenues for renal pathologies. Owing to their inherent kidney-targeting abilities, these nanomaterials can preferentially amass in diseased tissues, minimizing potential side effects on the rest of the body and highlighting an innovative perspective for the management of DKD. However, to ensure that the role of ROS-based nanomaterials is achieved, we need to pay attention to the biosafety, therapeutic efficacy, and targeting of the materials in the design process, and at the same time, we also need to dynamically monitor the changes of ROS *in vivo* to facilitate adaptation to disease states.

### 4.1 Biosafety

The potential toxicity of various biocatalytic and antioxidant nanostructures is a critical reason to impede their clinical applications. Research has indicated that polymer nanoparticles, lipid-based nanoparticles (LNPs), certain metal oxide nanoparticles, and carbon-based nanomaterials exhibit favorable biocompatibility. Polymer nanoparticles, comprising various synthetic and natural polymers such as polylactic acid (PLA) and polycaprolactone (PCL), can be utilized to formulate biocompatible nanoparticles. These polymers possess tunable degradation properties, contributing to the reduction of potential toxicity. Many polymer particles are sufficiently small (<20 nm) that they ought to cross the glomerular filtration barrier. In mice, a PEG-PLL carrier significantly enhanced the delivery of fluorescently tagged siRNA to the kidneys, compared with a naked siRNA control (44). LNPs are typically <100 nm in diameter, can be engineered to traverse the phospholipid cell membrane, and have very little toxicity and immunogenicity (45). Some metal oxide nanoparticles, such as silicon dioxide (SiO_2_) and iron oxide (Fe_2_O_3_), and carbon-based nanomaterials, including carbon nanotubes and graphene, can exhibit good biocompatibility under specific conditions. Of course, nanomaterials’ biocompatibilities are always associated with particle sizes, compositions, surface groups, degradability, and metabolic products, and there is an urgent need to fully disclose the toxicity of different types of biocatalytic and antioxidant nanostructures in the long term in animal-level observations to provide a comprehensive evaluation on their biocompatibilities.

Ensuring the biosafety of nanomedicines is fundamental for their biomedical utilization and subsequent clinical translation. While antioxidants at low concentrations provide a protective shield against cellular oxidative damage, elevated concentrations might paradoxically foster ROS production and incite cytotoxic effects (46). A comprehensive assessment of the safety profile of nanomedicines, especially in the context of renal diseases, is imperative to ascertain potential renal impairments or dysfunctions in other organs. The toxicity of NPs is intrinsically linked to their biophysical characteristics, encompassing dimensions, surface area, electrostatic charge, and aggregation state. These attributes influence the dispersal and accumulation of NPs across organ systems and modulate their molecular interplay with an array of proteins and macromolecules (47). Predominant toxic mechanisms stemming from NPs encompass ROS induction, DNA interference, protein structure and functionality perturbations, and membrane integrity disruptions (48). Generally, smaller particles manifest heightened chemical and biological reactivity due to their augmented surface-to-volume ratio. A salient toxicological feature of NPs revolves around amplified ROS production, stemming from the elevated reactivity of nanomaterials—a phenomenon evidenced across diverse nanomaterial categories (49).

Upon systemic introduction, primarily through intravascular routes, NPs exhibit a propensity for deposition within pivotal organs, including the lungs, liver, and kidneys, potentially instigating localized organ-specific toxic effects. The kidneys, serving as cardinal conduits for eliminating metabolic by-products, invariably encounter a multitude of NPs, with diminutive particles particularly prone to renal clearance. This predisposes the renal system to NP accumulation and potential adverse ramifications (6). It is paramount to recognize that ROS-based nanomaterial strategies targeting renal afflictions, most notably DKD, are developing. Biosafety concerns loom large, hindering their prospective advancements. *In-vitro* evaluations reveal that specific nanoparticles, such as those derived from platinum, silver, titanium dioxide, and silicon, can deleteriously impact the renal system, particularly affecting glomerular and tubular cells. Complementing this, *in-vivo* investigations elucidate structural distortions within typical renal units and compromised renal functionalities following exposure to these nanoparticles (50). Hence, as we forge ahead in harnessing ROS-based nanomaterials for DKD interventions, meticulous strategies should be delineated for nanomaterial design, ensuring minimized toxicity and optimal biosafety.

### 4.2 Therapeutic efficacy

The activity and stability of ROS-based nanomaterials, particularly during enzymatic catalysis, can be influenced by factors including particle size, composition, concentration, and environmental parameters such as redox states and pH values. Hence, rigorous examinations of their composition, structure, and other physical and chemical attributes—like size, surface charge, shape, and rigidity—as well as their delivery and administration methods are imperative to achieve the desired pharmacokinetics, target-specific drug delivery, optimal efficacy, and minimized toxicity. Notably, while the oral administration of antioxidant nanomaterials has exhibited subpar effectiveness, intravenous administration encounters a myriad of physiological and biological barriers, including the mononuclear phagocyte system and vascular flow constraints, which can significantly impede target-specific bioavailability (51).

At present, some emerging drugs for the treatment of DKD also have significant therapeutic development prospects, such as antirenal fibrosis drugs (52), activators of the Nrf2 pathway (53), endothelin-1 receptor antagonists (54), and so on. Targeted renal therapy may significantly improve the therapeutic effect by combining appropriate drugs and nanomaterials. Furthermore, it is essential to optimize further the reliability of endogenous stimulus-responsive modalities, including pH (55), temperature (56), and enzymes (57). In recent years, stimuli-responsive nanomaterials have provided an important strategy to maximize the treatment efficacy and reduce the damage to normal tissues, which is another important direction for treating DKD.

### 4.3 Kidney targeting

It is imperative to achieve efficient delivery to renal tissue to augment the therapeutic potential of nanomedicine for DKD patients. Current renal-targeting nanodrug delivery systems are tailored for various crucial targets, including glomerular endothelial cells (58), glomerular basement membrane (59), podocytes (60), glomerular mesangial cells (61), and renal tubules (62) (**Table 1**). Despite these advancements, the successful delivery of nanodrugs to renal tissues still needs to be improved. Efforts have been directed toward refining the size, morphology, and surface characteristics of nanodrugs. Nonetheless, enhancing the renal-targeting efficiency of these nanodrugs in clinical settings continues to be challenging, mainly due to the unique structural features of the kidney and the intricacies of the glomerular filtration barrier.

**Table 1:** Targeting drug delivery to the kidney.

Target cells	Materials	References
Glomerular endothelial cells	Liposomes	(63)
	Nanoparticle	(61)
Glomerular basement membrane	CDP-nanoparticles	(59)
Podocytes	Antibody-modified carrier	(64)
	Liposomes	(65)
Glomerular mesangial cells	AuNPs	(66)
Renal tubules	Polymers	(67)
	PLGA, ceria	(68)
	Chitosan	(69)

It is widely accepted that NPs with diameters of 3 nm or less permeate tissues non-specifically. Those under 5.5 nm undergo renal clearance, while NPs larger than 15 nm typically resist renal excretion (70). Intriguingly, NPs of varying sizes demonstrate an affinity for different renal compartments: approximately 10 nm-sized NPs localize within the glomerular basement membrane lattice, 30–40 nm NPs are found within the spaces between podocyte foot processes (71), and 60–70 nm NPs distribute across the gaps in the endothelial cells of the peritubular capillaries (72). Mesoscale nanoparticles (MNPs) are nanoparticles with a diameter exceeding 100 nm. Williams et al. synthesized MNPs with a diameter of approximately 400 nm, which avoided uptake by MPS organs and selectively accumulated in the proximal tubules (73). The accumulation efficiency in the kidneys was 26 times higher than in other organs. Histological evidence indicates that the nanoparticles predominantly localize to the basolateral side of the renal tubules rather than the apical side, suggesting that the accumulation of MNPs in the kidneys is facilitated by endocytosis of peritubular capillary endothelial cells. However, the physiological mechanisms still require further investigation. This glomerular filtration-independent delivery strategy may offer a novel approach to the treatment of tubular renal diseases. Beyond merely size and charge, the glomerular filtration propensity of nanoparticles is also influenced by their geometric configuration and flexibility. For instance, while the glomerulus typically resists filtering spheroidal albumin molecules (approximately 50 kDa in size) (74), more elongated molecules, like tannin and hyaluronan—despite possessing comparable molecular weights and charges—can traverse the glomerulus similar to albumin. Notwithstanding these hurdles, there remains a pressing need to innovate biomaterials proficient in targeting renal tissues, primarily through the challenges presented by the glomerular filtration barriers.

In the extensive research field of kidney-targeted nanomaterials, many review articles comprehensively and thoroughly investigated this topic. These reviews covered various aspects, including the types of materials, preparation methods, application areas, and potential biological effects. Chen et al. summarized the physiological and anatomical properties of the kidney and the biological and physicochemical characteristics of drug delivery systems, which affect the ability of drugs to target the kidney, and highlighted the prospects, opportunities, and challenges of nanotechnology in the therapy of kidney diseases (11). Huang et al. presented recent developments of nanoparticles designed for kidney-targeted delivery and briefly introduced targeting strategies for the kidney (75). Alallam et al. highlighted the features of nanomaterials, including NPs and the corresponding hydrogels, in overcoming various barriers to drug delivery to the kidneys. The most common delivery sites and strategies of kidney-targeted drug delivery systems were also discussed (76). Compared with these reviews, the uniqueness of this review lies in its focus on the application of kidney-targeted nanomaterials in DKD. While previous reviews primarily concentrated on the properties of materials and their impact on the kidneys, our review is centered on the potential application of these nanomaterials in patients with DKD, characterized by a highly oxidative stress environment.

### 4.4 Detection and monitoring of ROS

A significant challenge in the clinical development of nanomaterials pertains to redox pathology. Diseases associated with oxidative stress often exhibit heterogeneity in various oxidative species’ types, concentrations, and metabolism. Crucially, the redox state evolution, commencing from the onset of the disease, predominantly determines the requisite dosage and periodicity of antioxidants. Consequently, there is an imperative need for specific and sensitive monitoring of ROS dynamics to enrich our comprehension of the pathological significance of ROS in organisms. A widely employed measurement technique is electron paramagnetic resonance (EPR), which is alternately known as electron spin resonance (ESR) or electron magnetic resonance (EMR) spectroscopy. EPR spectrophotometers are adept at determining ROS *in vivo*, offering high specificity and sensitivity in ROS detection. Its capability for *in-vivo* ROS detection precludes potential artificial interference. Notably, EPR spectrophotometry can reliably detect free radicals, even at minuscule concentrations (e.g., 1 µM) (77).

However, the specificity of free radical detection through spin trapping may occasionally be indistinct, rendering EPR-based detection of free radical complex, particularly given the transient lifespan and scarce production of free radicals in living tissues. Although nascent technologies, such as fluorescence and photoacoustic imaging, facilitate ROS detection with enhanced sensitivity and specificity, there exists a pressing need to fine-tune imaging probes concerning their biocompatibility, photostability, solubility, organ specificity, imaging depth, and adaptability to biological milieu. At present, the meticulous quantification and analysis of diverse ROS pose a considerable challenge. This underscores the exigency to pioneer advanced techniques capable of discerning and quantifying varied free radicals, thus paving the way for the clinical application of antioxidant therapies. Moreover, specific strategies promise to circumvent the pitfalls associated with conventional antioxidant analyses, furnishing more dependable metrics of nano-antioxidant bioactivity. In summation, a multifaceted detection approach would offer an exhaustive assessment of the antioxidant potential of nanomaterials.

## 5 Conclusion and perspectives

ROS-based nanomaterials have unique properties such as targeting, stability, and adjustable ROS levels, making them competitive candidates for the treatment of DKD. In this review, we discuss the application of different types of ROS-based nanomaterials for the treatment of DKD, namely, ROS-scavenging nanomaterials, ROS-related nanodrug delivery materials, and ROS-responsive nanomaterials, which provide indications for better treatment of DKD.

However, the role of ROS nanomaterials is affected by various factors such as their composition, structure, and physicochemical properties, so we should pay attention to the therapeutic effect, biosafety, targeting, and dynamic monitoring of ROS in the design process. We believe that with the deepening of our understanding of renal pathology and the development of nanotechnology applications, ROS-based nanomaterials have great potential in the treatment of DKD. Despite the promising potential of nanomedicine in the treatment of DKD, the lack of nanoformulations advancing to clinical trials may be attributed to a combination of factors. Technical challenges in nanoparticle design and formulation, concerns regarding safety and efficacy profiles, and barriers to clinical translation could be hindering the progression of nanotherapeutics for DKD. Further research and collaboration are needed to address these challenges and accelerate the development of nanoformulations toward clinical trials for DKD treatment.

## Author contributions

QH: Writing – original draft. JT: Writing – original draft. YD: Writing – review & editing. FL: Writing – review & editing.
